# Further validation of the Multidimensional Fatigue Inventory in a US adult population sample

**DOI:** 10.1186/1478-7954-7-18

**Published:** 2009-12-15

**Authors:** Jin-Mann S Lin, Dana J Brimmer, Elizabeth M Maloney, Ernestina Nyarko, Rhonda BeLue, William C Reeves

**Affiliations:** 1 Chronic Viral Diseases Branch, National Center for Zoonotic, Vector-borne and Enteric Diseases, Centers for Disease Control and Prevention, Mail Stop A-15, 1600 Clifton Rd, NE, Atlanta, GA, USA; 2 Department of Global Health, Rollins School of Public Health, Emory University, Atlanta, GA, USA; 3 Department of Health Policy and Administration, The Pennsylvania State University, University Park, PA, USA

## Abstract

**Background:**

The Multidimensional Fatigue Inventory (MFI-20) was developed in 1995. Since then, it has been widely used in cancer research and cancer-related illnesses but has never been validated in fatiguing illnesses or in a large US population-selected sample. In this study, we sought to examine the reliability and validity of the MFI-20 in the population of the state of Georgia, USA. Further, we assessed whether the MFI-20 could serve as a complementary diagnostic tool in chronically fatigued and unwell populations.

**Methods:**

The data derive from a cross-sectional population-based study investigating the prevalence of chronic fatigue syndrome (CFS) in Georgia. The study sample was comprised of three diagnostic groups: CFS-like (292), chronically unwell (269), and well (222). Participants completed the MFI-20 along with several other measures of psychosocial functioning, including the Medical Outcomes Survey Short Form-36 (SF-36), the Zung Self-Rating Depression Scale (SDS), and the Spielberger State-Trait Anxiety Inventory (STAI). We assessed the five MFI-20 subscales using several criteria: inter-item correlations, corrected item-total correlations, internal consistency reliability (Cronbach's alpha coefficients), construct validity, discriminant (known-group) validity, floor/ceiling effects, and convergent validity through correlations with the SF-36, SDS, and STAI instruments.

**Results:**

Averaged inter-item correlations ranged from 0.38 to 0.61, indicating no item redundancy. Corrected item-total correlations for all MFI-20 subscales were greater than 0.30, and Cronbach's alpha coefficients achieved an acceptable level of 0.70. No significant floor/ceiling effect was observed. Factor analysis demonstrated factorial complexity. The MFI-20 also distinguished clearly between three diagnostic groups on all subscales. Furthermore, correlations with depression (SDS), anxiety (STAI), and functional impairment (SF-36) demonstrated strong convergent validity.

**Conclusions:**

This study provides support for the MFI-20 as a valuable tool when used in chronically unwell and well populations. It also suggests that the MFI-20 could serve as a complementary diagnostic tool in fatiguing illnesses, such as CFS.

## Background

Fatigue is a common symptom associated with numerous acute and chronic illnesses. Fatigue is one of the most frequent symptoms reported to physicians; between 7% and 45% of primary care consultations involve fatigue [1,2]. High levels of fatigue negatively affect quality of life for patients with cancer, Parkinson's disease, multiple sclerosis, and persons with less well-understood illnesses such as chronic fatigue syndrome (CFS) and fibromyalgia [3-6]. The fatigue associated with various conditions is generally not alleviated by rest and precludes normal mental and physical activities. Fatigue also often accompanies affective disorders [7]. Fatigue was significantly positively correlated with depression in patients with multiple sclerosis [8-10] and in patients with unexplained fatigue [11]. The national Canadian Community Health Survey reported that 36% of individuals with CFS were depressed [12], whereas a population-based study of CFS reported that 22% of individuals with CFS in Georgia had major depressive disorder, and 46% had anxiety disorders [13]. Roy-Byrne et al. [14] found that fatigued twins were more somatically preoccupied and anxious than non-fatigued twins. Optimal management of patients with fatiguing illnesses requires assessing the nature, frequency, severity, and duration of fatigue and evaluating effects of interventions on fatigue. Although several standardized instruments have been designed to evaluate fatigue, they have not been validated across illnesses in adults.

The Multidimensional Fatigue Inventory (MFI-20) was developed by a Dutch group in 1995 to measure fatigue severity [2]. The MFI-20 was first evaluated in a group of people with CFS, cancer patients, a healthy control group comprised of psychology and medical students, and a group of army recruits [2]. The MFI-20 showed good internal consistency (Cronbach's alpha > 0.80) for the general, physical, and mental fatigue dimensions, and adequate reliability for the reduced activity and motivation items (Cronbach's alpha > 0.65). Construct validity between the different test groups was significant at p < 0.001 for all five dimensions. Convergent validity between the MFI-20 and the Visual Analogue Scale (VAS) fatigue score for the group of cancer patients was significant for all subscales.

Validity and reliability of the MFI-20 have also been evaluated in several other non-US populations. These included patients with cancer [15-17], chronic fatigue [3,18], craniopharyngioma [19], myelodysplastic patients [20], thyroid disease, and a "not tired" control group [18].

Test-retest reliability of the MFI-20 has been reported in several European studies. Ericsson and Mannerkorpi validated the MFI-20 in 166 Swedish patients with fibromyalgia and chronic widespread pain [3]. Hagelin et al. [17] validated the MFI-20 in four groups composed of 584 Swedish subjects: palliative cancer patients, cancer patients receiving radiation therapy, noncancer outpatients, and a group of hospital staff. Gentile et al. [18] validated the MFI-20 in three groups of French subjects: tired, (82 subjects), moderately tired (36), and not tired (107). Finally, Schwarz et al. [21] published the population norms for the five MFI-20 subscales in a sample of 2,037 adult Germans. The crucial result of the Schwarz study was the quantification of age and sex dependency in fatigue.

In the United States, Schneider validated the MFI-20 in 97 rural oncology outpatients and in 45 spouses or first-degree female caregivers of male hemodialysis patients in northern and eastern Iowa [22,23]. To our knowledge, the MFI-20 has not been validated in persons from the US with fatiguing illnesses nor in a large US population-selected sample. The aims of the present study were: 1) to investigate the reliability and validity of the MFI-20 in chronically unwell and well persons; 2) and to assess whether the MFI-20 could serve as a complementary diagnostic tool in populations with fatiguing illnesses.

## Methods

The data came from a cross-sectional, population-based study investigating the prevalence of CFS in Georgia. Details of the source study have been previously published [24] but are summarized here. The Centers for Disease Control and Prevention (CDC) Institutional Review Board, as required by US Department of Health and Human Services regulations, approved the study. All participants provided informed consent.

### Study design and sample

The study was carried out in two phases between September 2004 and July 2005. Phase 1 involved a random-digit-dialing telephone survey to screen 19,381 adult residents (96% response) ages 18 to 59 from metropolitan, urban, and rural Georgia populations. Based on the 19,381 people from the household screening interview, 8,910 adults were randomly selected for detailed telephone interviews: 5,623 individuals completed the detailed telephone interview; 1,874 refused to participate; 141 were further confirmed to be ineligible; and 1,272 were excluded due to physical or mental inability to participate, inability to be contacted, language barriers, or because they had died. This yielded an overall response rate of 75%. Based on the detailed telephone interviews, study participants were classified into three groups:

1) CFS-like, characterized by severe fatigue lasting six months or longer that was not alleviated by rest, that caused substantial reduction in occupational, educational, social, or personal activities, and that was accompanied by at least four of the CFS case-defining symptoms.

2) Chronically unwell, having chronic (≥ six months) unwellness with or without fatigue, but not meeting the criteria for CFS.

3) Well.

In Phase 2, all 469 people with CFS-like illness were invited for clinical evaluation, and 292 (62%) participated. Of randomly selected chronically unwell participants, 286 (53%) completed the clinical evaluation. Finally, 223 individuals classified as well in the telephone interview completed clinical evaluations. They were matched to the CFS-like group based on residence (metropolitan, urban, rural), sex, race/ethnicity, and age (within three years). Overall, about 50% of invited respondents from all three groups completed the one-day clinical evaluation.

Participants completed the MFI-20 and other questionnaires during the clinical evaluation. This study involves data from 783 participants who completed the MFI-20 along with several other measures of psychosocial functioning, including the Medical Outcomes Survey Short Form-36 (SF-36), the Zung Self-Rating Depression Scale (SDS), and the Spielberger State-Trait Anxiety Inventory (STAI).

### Measures

#### MFI-20 Subscales

The MFI-20 comprises five subscales: general fatigue, physical fatigue, mental fatigue, reduced activity, and reduced motivation [2]. Each subscale includes four items with five-point Likert scales. General fatigue includes general statements about fatigue and decreased functioning and was designed to encompass both physical and psychological aspects of fatigue. Physical fatigue concerns physical sensations related to fatigue. Mental fatigue pertains to cognitive functioning, including difficulty concentrating. Reduced activity refers to the influence of physical and psychological factors on the level of activity. Reduced motivation relates to lack of motivation for starting any activity. Scores on each subscale range from 4 to 20, with higher scores indicating greater fatigue.

#### SF-36 Subscales

The SF-36 contains eight multi-item subscales: general health perceptions, physical functioning, role physical (role limitations due to physical problems), bodily pain, general mental health, vitality (vitality/energy/fatigue), role emotional (role limitations due to emotional problems), and social functioning. The number of response choices per item ranges from two to six. Each transformed subscale has a range from 0 to 100 (100 = optimal function) [25]. The SF-36 also yields two summary scores that reflect the two-dimensional factor structure underlying the eight subscales: a physical component summary (PCS) score and a mental component summary (MCS) score. PCS and MCS are a linear combination of eight SF-36 subscales, but PCS is predominantly based on the subscales physical functioning, role physical, bodily pain, and general health perceptions, and MCS is predominantly based on the scales mental health, role emotional, social functioning, and vitality (range 0-100, 100 = optimal) [26].

#### SDS Subscale

The SDS [27] includes 20 questions that quantify the severity of depression symptoms. Each item ranges from 1 (none or a little of the time) to 4 (most or all of the time). The raw SDS score is the sum of all 20 items and ranges from 20 to 80. Following standard practice, we converted raw SDS scores to a 100-point scale (SDS index) in which < 50 = normal, 50-59 = mild depression, 60-69 = moderate to marked depression, and ≥ 70 = severe depression.

#### STAI Subscales

The STAI [28] includes 40 questions with four possible responses to each. It was constructed as two subscales: 20 items to assess state anxiety, and another 20 to assess trait anxiety. State anxiety is defined as a transient, momentary emotional status that results from situational stress. Trait anxiety represents a predisposition to react with anxiety in stressful situations. Each subscale ranges from 20 to 80, with higher scores indicating higher anxiety. These two parts differ in the item wording, in the response format (intensity versus frequency), and in the instructions for how to respond. The STAI clearly differentiates between the temporary condition of state anxiety and the more general and long-standing quality of trait anxiety.

All four questionnaires were self-reported and self-administered by participants. The mean time taken to complete each questionnaire was five, nine, three, and four minutes for the MFI-20, SF-36, SDS, and STAI, respectively. The Flesch Reading Ease formula and a Flesch abstraction formula were applied. The measures are generally shown to be useful for respondents with a sixth grade reading level or below. The reading level of each respondent was assessed by the Wide Range Achievement Test reading subtest [29], and only 45 (6%) of respondents were below a sixth grade reading level.

### Statistical analysis

We used SAS version 9.1 (SAS Institute Inc, Cary, NC) for data analysis. Descriptive statistics (frequencies, percentages, means, standard deviations, and ranges) were generated to characterize the study sample in terms of socio-demographic parameters. We used several criteria to assess the subscale validity and reliability of the MFI-20.

Internal consistency of each of the five MFI-20 subscales was determined using three reliability tests: 1) inter-item correlation; 2) corrected-to-total (or item-total) subscale correlation; 3) and Standardized Cronbach's α coefficients (and item discrimination). The cutoff criteria for acceptance on reliability tests are as follows. First, item-total subscale correlations of not less than 0.30 and inter-item correlations of 0.30 to 0.70 were retained. Second, a fairly high reliability coefficient (Cronbach's α > 0.70) was required to assess the internal consistency reliability [30,31]. Floor/ceiling effects were considered significant if more than 15% of the subjects had either the lowest possible or highest possible score on the subscales [32]. A significant floor effect was expected in the well group.

As an indication of discriminant (known-group) validity, group differences in the five MFI-20 subscales were calculated using analyses of variance to examine the ability of the MFI-20 instrument to distinguish three groups: CFS-like, chronically unwell, and well. Using a Tukey correction, the alpha per test for each subscale was 0.01, for an overall alpha of 0.05. Two-way analyses of variance were performed to test the age and sex effects on the five MFI-20 subscales. Post-hoc analysis with Tukey p-value adjustment was performed for multiple subgroup comparisons.

To further assess construct validity of the subscales, an exploratory factor analysis was performed. A principle component analysis was used to extract factors. The obtained factors were rotated oblique using the Varimax procedure. A minimum eigenvalue of 1 was specified as the extraction criterion [33]. The desired criterion of factor loadings was set at 0.50 or above, slightly higher than the typical cutoff value of 0.40 [34].

Finally, the convergent validity of the MFI-20 was evaluated through comparisons of the MFI-20 with other instruments administered in the protocol. Pearson correlation coefficients were used to assess linear associations between the multi-item scales of SF-36, SDS, and STAI. We chose these instruments based on the association between fatigue and other measures on psychosocial functioning, such as health-related quality of life (measured by SF-36), depression (measured by SDS), and anxiety (measured by STAI) as well as the existing data from the source study.

The most valid SF-36 subscales for measuring physical health include the physical functioning, role physical, and bodily pain subscales and the physical component summary score [26]. The most valid SF-36 subscales for measuring mental health include the mental health, role emotional, and social functioning subscales and the mental component summary score [26]. For the concept of physical and mental health, we investigated correlations between MFI-20 subscales and physical and mental health as measured by the SF-36.

## Results

Data completeness was high, with only one missing response for the reduced activity subscale among all five subscales. This indicated that the MFI-20 was well-accepted in our study sample of chronically unwell and well people.

### Sample characteristics

Table 1 summarizes subscale validity and reliability analyses for the 783 participants who completed the MFI-20 questionnaire. Of these, 37% had been classified as CFS-like based on the detailed telephone interview, 34% were chronically unwell, and 28% were considered well. The participants had a mean age of 43, were primarily female (76%), white (70%), and from rural or urban areas (83%). Nearly 95% had completed at least a high school education. Nearly 38% were unemployed, self-employed (not working for pay), retired, laid off, disabled, or students. More than 60% of participants were married or cohabitating. More than half of participants had a household income equal to or higher than the Georgia median income level of $42,679.

**Table 1:** Characteristics of the study sample

Characteristic	CFS-like (n = 292, 37.29%)	Chronically Unwell (n = 269, 34.36%)	Well (n = 222, 28.35%)
Age, yrs, Mean (SD)	43.73 (9.87)	43.05 (11.24)	43.68 (9.96)
Sex****			
Female	243 (83.22%)	170 (63.43%)	184 (82.51%)
Male	49 (16.78%)	98 (36.57%)	39 (17.49%)
Race**			
Black	65 (22.26%)	84 (31.34%)	48 (21.52%)
White	208 (71.23%)	173 (64.55%)	170 (76.23%)
All Others	19 (6.51%)	11 (4.10%)	5 (2.24%)
Geographic Areas			
Metropolitan	43 (14.73%)	56 (20.90%)	33 (14.80%)
Urban	108 (36.99%)	78 (29.10%)	81 (36.32%)
Rural	141 (48.29%)	134 (50.00%)	109 (48.88%)
Educational Status***			
< High School	23 (7.88%)	15 (5.60%)	7 (3.14%)
High School Graduate/GED	75 (25.68%)	52 (19.40%)	33 (14.80%)
Trade, Technical, or Vocation School after High School	30 (10.27%)	44 (16.42%)	24 (10.76%)
Some College	68 (23.29%)	49 (18.28%)	47 (21.08%)
2-yr College Graduate or Higher	95 (32.53%)	107 (39.93%)	112 (50.22%)
Missing	1 (0.34%)	1 (0.37%)	0 (0.00%)
Marital Status			
Married/living together	184 (63.01%%)	168 (62.69%)	151 (67.71%)
Single/widowed/divorced/seperated	107 (36.64%)	100 (37.31%)	72 (32.29%)
Missing	1 (0.34%)	0 (0.00%)	0 (0.00%)
Employment Status****			
Full Time: >= 30 hours/week	161 (55.14%)	167 (62.31%)	159 (71.30%)
Part Time: < 30 hours/week	31 (10.62%)	26 (9.70%)	23 (10.31%)
Self-employed	2 (068%)	0 (0.00%)	2 (0.90%)
Not employed	16 (5.48%)	23 (8.58%)	3 (1.35%)
Retired	2 (0.68%)	6 (2.24%)	9 (4.04%)
Laid off	4 (1.37%)	6 (2.24%)	2 (0.90%)
Disabled	45 (15.41%)	17 (6.34%)	2 (0.90%)
Homemaker	20 (6.85%)	14 (5.22%)	17 (7.62%)
Student	10 (3.42%)	9 (3.36%)	6 (2.69%)
Missing	1 (0.34%)	0 (0.00%)	0 (0.00%)
Income*			
>= GA Median Income	146 (50.00%)	141 (52.61%)	142 (63.68%)
< GA Median Income	133 (45.55%)	117 (43.66%)	75 (33.63%)
Missing	13 (4.45%)	10 (3.73%)	6 (2.69%)

All values are No. (%) unless other indicated. ^†^Column percentage

### Associations of age and sex with MFI-20 subscale scores

Only the physical fatigue subscale score differed significantly by both age (p = 0.0024) and sex (p = 0.0015). Reduced activity (p = 0.0078) and reduced motivation (p = 0.0112) scores differed significantly between age groups. General fatigue (p = 0.0003) and mental fatigue (p = 0.0272) scores were significantly worse in females than in males. The interaction between age and sex was not significant in any of the MFI-20 subscales. Although only three of the five MFI-20 subscales were significantly different by sex, descriptive statistics of all the subscales were summarized for females and males (Table S1 and Table S2, Additional file 1).

For subscales with significant age or sex effects, we estimated partial correlations controlling for sex and age, respectively (Table 2). This had negligible effects on the correlations between the physical fatigue, reduced activity, and reduced motivation subscales.

**Table 2:** Correlations among MFI-20 subscales and their partial correlations controlled for age or sex.

Age Effect			
	
	Physical Fatigue	Reduced Activity	Reduced Motivation
Physical Fatigue		0.6777	0.6301
Reduced Activity	***0.6738***		0.6893
Reduced Motivation	***0.6255***	***0.6855***	

**Sex Effect**			
	
	**Physical Fatigue**	**General Fatigue**	**Mental Fatigue**

Physical Fatigue		0.7392	0.4933
General Fatigue	***0.7356***		0.5940
Mental Fatigue	***0.4891***	***0.5904***	

Note: upper right triangle: Pearson correlations; lower left triangle (numbers in Italic and bold): partial correlations (controlled for sex and age, respectively). All p-values are < 0.0001.

### Reliability

Table 3 summarizes the results of three reliability tests for the five MFI-20 subscales. There was no item redundancy; inter-item correlations averaged 0.56 (range 0.46-0.69) for general fatigue, 0.52 (range 0.44-0.61) for physical fatigue, 0.53 (0.41-0.66) for reduced activity, 0.38 (0.17-0.56) for reduced motivation, and 0.61 (0.53-0.66) for mental fatigue. Corrected item-total correlations were higher than 0.30 for all five MFI-20 subscales. The values for standardized Cronbach's α for the five MFI-20 scales were: general fatigue: 0.83; physical fatigue: 0.81; reduced activity: 0.82; reduced motivation: 0.71; and mental fatigue: 0.86. These values were greater than the suggested criteria value of 0.70 for acceptable reliability.

**Table 3:** MFI-20 scale item characteristics and internal consistency reliabilities.

			Inter-item correlation	Corrected-to-total correlation	Coefficient α if item deleted	Standardized Cronbach's
			
	Mean	SD	Mean (Range)	Range	Range	α
General Fatigue	12.90	4.68	0.56 (0.46-0.69)	0.59-0.70	0.77-0.84	0.83
Physical Fatigue	10.85	4.36	0.52 (0.44-0.61)	0.59-0.67	0.75-0.79	0.81
Reduced Activity	9.25	4.16	0.53 (0.41-0.66)	0.51-0.71	0.75-0.84	0.82
Reduced Motivation	9.58	3.90	0.38 (0.17-0.56)	0.33-0.62	0.57-0.75	0.71
Mental Fatigue	10.95	4.54	0.61 (0.53-0.66)	0.68-0.75	0.81-0.84	0.86
Total Fatigue Score	53.53	17.93	0.40 (0.11-0.68)	0.33-0.77	0.92-0.93	0.93

### Relationships among five MFI-20 subscales

Pairwise correlations between the MFI-20 subscales ranged from 0.49 to 0.74. Although the subscales are strongly related to each other, it is unclear whether an overall summary component of the MFI-20 is appropriate. Factor analysis confirmed that overall summary components accounted for 70% of the reliable variance in the five subscales. The total scale with 20 items yielded a Cronbach's α coefficient of 0.93, which is consistent with the result from the Gentile study [18].

The factor analysis solution was complex, with multiple loadings of items having factor-loading values > 0.50 across five factors (Table 4). However, the first factor, which explained 20% of the variance in the 20 items of the MFI-20, was dominated by general fatigue and physical fatigue. Six items (four physical fatigue, one general fatigue, and one reduced activity) loaded on the first factor (loadings from 0.54 to 0.83). The second factor was comprised solely of all four mental fatigue items (loadings from 0.71 to 0.81), which explained 15% of the variance in the 20 items of the MFI-20. Three of the reduced activity items fell nicely (loading from 0.52 to 0.71) on the third factor. The fourth factor was loaded by three of the general fatigue items (loading from 0.52 to 0.71) and two of the reduced motivation items (loading 0.58 and 0.64). The remaining two reduced motivation items fell nicely on the fifth factor.

**Table 4:** Factor analysis of 20 MFI item responses.

	Five Factors				
	**1**	**2**	**3**	**4**	**5**

**General Fatigue**					
I feel fit	*.83*				
I feel tired				.71	
I feel rested				.52	
I tired easily				.61	
**Physical Fatigue**					
Physically I feel I am in an excellent condition	.81				
Physically I feel I am in a bad condition	.67				
Physically I can take on a lot	.56				
Physically I feel only able to do a little	.54				
**Reduced Activity (Vigor)**					
I think I do very little in a day			.84		
I think I do a lot in a day			.78		
I get little done			.72		
I feel very active	*.68*				
**Reduced Motivation**					
I have a lot of plans					.89
I feel like doing all sorts of nice things					.53
I dread having to do things				.64	
I don't feel like doing anything				.58	
**Mental Fatigue (Cognition)**					
When I am doing something, I can keep my thoughts on it		.81			
I can concentrate well		.81			
My thoughts easily wander		.75			
It takes a lot of effort to concentrate on things		.71			
Total % of Variance Explained	20.10	15.18	14.10	13.40	6.87

Note: Factor loadings less than 0.5 were not listed in the table. The numbers in bold indicated the largest factor loading of the item loaded on different factor components.

Kaiser-Meyer-Olkin (KMO) = 0.938, p-value for Bartlett's Test of Sphericity is < 0.001. Cumulative % of Variance Explained is 70%.

### Discriminant (known-group) validity: MFI-20 subscale differences between three groups

The CFS-like, chronically unwell, and well groups had significantly different mean values (p < 0.0001) for all the MFI-20 subscales (Table 5). All subscales appeared to discriminate between groups, but the degree to which they discriminated varied. The CFS-like group had higher scores in all the subscales compared to the chronically unwell group (average mean difference = 2.90; range of mean difference: 2.26 points (reduced activity) - 3.54 points (general fatigue). Compared to the well group, the CFS-like group had subscale scores that were, on average, 6.01 points higher. Mean differences between these groups ranged from 4.56 points (reduced activity) to 7.96 points (general fatigue), whereas the chronically unwell group scored, on average, 3.11 points higher in the five subscales than the well group.

**Table 5:** Descriptive statistics for the five MFI-20 scales by subgroups

	All	CFS-like	Chronically Unwell	Well
**General Fatigue**				
**Mean**	**12.90**	**16.38**	**12.84**	**8.42**
**SD**	**4.68**	**2.73**	**3.93**	**3.59**
25%	9.00	15.00	10.00	6.00
Median	14.00	17.00	13.00	8.00
75%	17.00	18.00	16.00	11.00
Range	4-20	6-20	4-20	4-20
% at floor	3.45	0	1.49	10.31
% at ceiling	6.13	13.01	3.36	0.45
**Physical Fatigue**				
**Mean**	**10.85**	**13.63**	**10.39**	**7.77**
**SD**	**4.36**	**3.79**	**3.76**	**3.36**
25%	7.00	11.00	8.00	5.00
Median	11.00	14.00	10.00	7.00
75%	14.00	16.00	13.00	10.00
Range	4-20	4-20	4-20	4-19
% at floor	6.39	0.34	5.60	15.25
% at ceiling	2.81	6.51	1.12	0
**Reduced Activity**				
**Mean**	**9.25**	**11.32**	**9.06**	**6.76**
**SD**	**4.16**	**4.37**	**3.75**	**2.67**
25%	6.00	8.00	6.00	5.00
Median	8.00	11.00	8.00	6.00
75%	12.00	15.00	12.00	8.00
Range	4-20	4-20	4-20	4-16
% at floor	11.49	3.77	8.96	24.66
% at ceiling	2.43	5.14	1.49	0
**Reduced Motivation**				
**Mean**	**9.58**	**11.95**	**9.29**	**6.82**
**SD**	**3.90**	**3.53**	**3.35**	**2.91**
25%	6.00	9.50	7.00	4.00
Median	9.00	12.00	9.00	6.00
75%	12.00	14.00	11.00	8.00
Range	4-20	4-20	4-20	4-20
% at floor	11.49	1.37	10.07	26.46
% at ceiling	0.77	1.37	0.37	0.45
**Mental Fatigue**				
**Mean**	**10.95**	**13.77**	**10.98**	**7.23**
**SD**	**4.54**	**3.77**	**4.00**	**3.07**
25%	7.00	11.50	8.00	4.00
Median	11.00	14.00	11.00	7.00
75%	14.00	17.00	14.00	9.00
Range	4-20	4-20	4-20	4-20
% at floor	9.96	1.37	6.34	25.56
% at ceiling	3.70	7.19	2.61	0.45

We observed a floor/ceiling effect in all the MFI-20 subscales in the well group, except for general fatigue, as expected. No floor/ceiling effects were detected in the CFS-like and chronically unwell groups. There were no floor/ceiling effects in the whole study sample (Table 5).

### Convergent validity: relationships to functional impairment, depression, and anxiety

We calculated correlations between fatigue subscales and subscales measuring functional impairment (SF-36), depression (SDS), and anxiety (STAI) to evaluate convergent validity in the overall sample (Table 6). The MFI-20 subscales were substantially correlated with the eight SF-36 subscales (average: r = -0.53; range of absolute values of correlations: |r| = 0.34 - 0.83). All MFI-20 subscales were most strongly correlated [35] with the SF-36 subscales measuring vitality (average: r = -0.68; range of |r|: 0.57 - 0.83), followed by general health perception (average: r = -0.59; range of |r|: 0.48 - 0.71), and social functioning (average: r = -0.54; range of |r|: 0.50 - 0.59).

**Table 6:** Convergent Validity: Pearson Correlation Coefficients between the MFI-20, SF-36, SDS, and STAI^† ^in overall sample.

	MFI-20					
	
	General Fatigue	Physical Fatigue	Reduced Activity	Reduced Motivation	Mental Fatigue	Total Score
SF-36						
Physical Functioning	-0.496	**-0.643**	**-0.522**	-0.490	-0.376	**-0.564**
Role Physical	**-0.591**	**-0.589**	-0.464	**-0.502**	-0.429	**-0.599**
Bodily Pain	-0.498	**-0.546**	-0.386	-0.390	-0.338	-0.486
**Social Functioning**	**-0.553**	**-0.535**	**-0.506**	**-0.592**	**-0.500**	**-0.645**
Mental Health	**-0.547**	-0.461	-0.436	**-0.564**	**-0.550**	**-0.631**
Role Emotional	-0.462	-0.409	-0.379	-0.435	-0.480	**-0.530**
**Vitality**	**-0.825**	**-0.689**	**-0.582**	**-0.708**	**-0.574**	**-0.811**
General Health	**-0.659**	**-0.713**	**-0.533**	**-0.562**	-0.479	**-0.672**
PCS^‡^	**-0.540**	**-0.671**	-0.492	-0.450	-0.327	**-0.544**
MCS^‡^	**-0.563**	-0.448	-0.455	**-0.589**	**-0.563**	**-0.653**
						
SDS						
SDS Index	**0.626**	**0.541**	0.498	**0.649**	**0.608**	**0.718**
						
STAI						
State-Anxiety Score	0.413	0.352	0.336	0.457	0.469	**0.503**
Trait-Anxiety Score	**0.519**	0.421	0.423	**0.558**	**0.563**	**0.620**

All p-values for pairwise Pearson correlations are less than 0.0001.

Absolute correlation coefficients of 0.5 to 1.0 [35] are considered high correlations, in bold.

^† ^Multidimensional Fatigue Inventory (MFI-20), the Medical Outcomes Survey Short Form-36 (SF-36), the Zung Self-Rating Depression Scale (SDS), and the Spielberger State-Trait Anxiety Inventory (STAI)

^‡ ^PCS: Physical Component Summary; MCS: Mental Component Summary

As expected, all five MFI-20 subscales were significantly correlated with depression, anxiety, and functional impairment. However, the correlations with depression and anxiety were generally lower (average: r = 0.50; range of r = 0.34-0.65) than correlations with functional impairment (the SF-36 subscales). The highest correlations were found between MFI-20 subscales and measurement of depression (SDS index) (average: r = 0.58; range of r = 0.50 - 0.65).

#### Conceptual relationship: Mental and Physical

The general fatigue subscale of the MFI-20 was associated with both physical and mental health, based on strong correlations (|r| >=0.5) with functional impairment as measured by the SF-36 subscales (except for physical functioning, bodily pain, and role emotional), and both the physical component summary score and mental component summary score. General fatigue was also highly associated with the SDS index and the STAI trait-anxiety subscale.

The physical fatigue subscale of the MFI-20 was highly correlated (|r| >=0.5) with several subscales of the SF-36 that measure predominantly physical health (physical functioning, role physical, bodily pain, social functioning, vitality, general health) and the physical component summary score but not the mental component summary score (Table 6). Physical fatigue was also highly correlated with the SDS index score measuring depression.

The mental fatigue subscale of the MFI-20 was highly correlated with several subscales of the SF-36 that measure predominantly mental health (social functioning, mental health, and vitality subscales), as well as the mental component summary score. The mental fatigue subscale was also associated with depression (SDS index) and trait anxiety (STAI).

The reduced activity subscale of the MFI-20 was highly correlated (|r| >=0.5) with several SF-36 subscales (physical functioning, social functioning, vitality, and general health perception). The reduced motivation subscale of the MFI-20 was highly correlated with many SF-36 subscales (role physical, social functioning, mental health, and vitality) as well as the mental component summary measure, but not the physical component score. Reduced activity was also correlated with depression (SDS index) and anxiety (STAI trait-anxiety).

We examined the total fatigue score of the MFI-20 in relation to other instruments. Total fatigue was highly correlated with all SF-36 subscales except for bodily pain, and was correlated with the physical component summary score and mental component summary score, as well as SDS index and state-anxiety and trait-anxiety subscales (STAI). The total fatigue score of the MFI-20 was highly consistent and demonstrated the highest correlations with other questionnaires.

#### Relationships to depression, anxiety, and functional impairment among classification groups

In the CFS-like group, the SF-36 subscale scores were highly correlated with the MFI-20 subscales for general fatigue, physical fatigue, reduced activity, and reduced motivation but not with mental fatigue. Also in this group, depression (SDS index) was highly correlated with reduced motivation (r = 0.50) but only moderately correlated with other subscales of the MFI-20. In general, the scores of the STAI correlated with all five MFI-20 subscale scores. The trait-anxiety score of the STAI had stronger correlations than state-anxiety with the MFI-20 subscale scores (Table S3, Additional file 1).

For the chronically unwell and well groups, depression and anxiety correlated with all five MFI-20 subscales. The bodily pain subscale and the physical component summary scores of the SF-36 did not correlate with the mental fatigue subscale of the MFI-20. Depression, as measured by the SDS index, correlated with all the MFI-20 subscales. The correlations between bodily pain of the SF-36 and activity fatigue (reduced activity or reduced motivation) are not statistically significant (Table S4 and Table S5, Additional file 1).

## Discussion

This study greatly extends previous research with the MFI-20 in several ways. The first objective of this study was to assess reliability and validity of the MFI-20 in chronically unwell and well groups identified from metropolitan, urban, and rural populations in the state of Georgia. The MFI-20 was well-accepted in our sample of unwell and well people. Low to moderate inter-item correlations indicated no item redundancy. Corrected item-total correlations for all MFI-20 subscales were all in an acceptable range. The MFI-20 item subscales exhibited adequate internal consistency reliability with Cronbach's α coefficients ranging from 0.72 to 0.86, which is consistent with results from previous studies [2,17,21,36]. We found no significant floor/ceiling effects in the whole study sample.

With respect to validity, the results of factor analysis of the MFI-20 in a sample of unwell and well people provide additional support for the five-factor structure of the MFI-20 [2]. As previously noted, however, some factors are highly correlated, and several items would have loaded on more than one factor had the paths not been constrained. In addition to forming its own factor component, one of the general fatigue subscale items loaded on the same factor with items of the physical fatigue subscale because it provides information about physical fitness. This general fatigue subscale item may be considered along with the physical fatigue subscale to assess fatigue scores in populations with fatiguing illnesses. The results also showed that a total fatigue summary score is a valid summary score for people with fatiguing illnesses.

In a further examination of known-group comparison for construct validity, all five MFI-20 subscales distinguished clearly between our three study groups. The magnitude of the mean group differences in the MFI-20 subscales is greater than the generic minimal clinically important difference (MCID) of two points across the pre- and post-radiotherapy comparison and occupational productivity anchor [37]. People with CFS-like illness had several higher fatigue and activity subscale mean scores that were both statistically and clinically significant (an average of three points higher) than those who were chronically unwell but did not have CFS-like illness. These differences were more exaggerated (six points higher, on average) when the CFS-like group was compared to the well group with respect to these subscales. As expected, those who were chronically unwell also had fatigue and activity subscale scores that were both statistically and clinically significant (three points higher than well people).

The MFI-20 subscales exhibited adequate convergent validity with other instruments. The general fatigue subscale of the MFI-20 is highly correlated with the functioning subscales of the SF-36, SDS depression, and the trait anxiety subscale of the STAI. This confirms that the general fatigue subscale represents both physical and psychological aspects of fatigue. Physical fatigue represents the physical sensation related to fatigue, which is validated by the substantial associations with physical functioning, role physical, bodily pain, social functioning, vitality, general health perception, and physical component summary measure. Reduced activity refers to the influence of both physical and psychological factors on the level of activity. Reduced motivation refers to the psychological experience of feeling unable to start an activity [38]. Finally, mental fatigue, which originally measures cognitive functioning such as difficulty concentrating, reflects the "mental health" concept of fatigue, which is validated by the associations with social functioning, mental health, and vitality as well as the mental component summary measure.

Our study showed that sex and age exert effects on several MFI-20 subscales. Compared to males, females had slightly higher mean scores for subscales measuring general fatigue, physical fatigue, and mental fatigue. This confirms previous findings of sex differences in mean scores of fatigue scales [21,39], and age-associated increases in mean scores in physical fatigue, reduced activity, and reduced motivation [18].

We showed that the five MFI-20 subscales were highly correlated with functional impairment, depression, and anxiety in the overall sample. Breslin et al. [40] showed that depression correlated with the general fatigue and mental fatigue subscales of the MFI-20 but not with physical fatigue in patients with chronic obstructive pulmonary disease (COPD). Schwarz et al. [21] showed that fatigue is correlated with hospital anxiety and depression scale (HADS) and the global quality-of-life scale.

Our CFS-like group provides the opportunity to examine the convergent validity of the MFI-20 with other measurements among people with fatiguing illness. In the CFS-like group, additional support for the validity of the MFI-20 is provided by the insignificant-to-moderate correlations between the SF-36 subscales and mental fatigue of the MFI-20. This indicates that mental fatigue is only partly measured by the SF-36 among individuals with CFS-like illness. Depression is moderately correlated with several subscales of the MFI-20. We also showed low correlations between state-anxiety of the STAI and general fatigue, physical fatigue, and reduced activity of the MFI-20. Therefore, the additional information provided by the MFI-20 may deepen our insight into functional impairment, depression, and anxiety in fatiguing illnesses.

### Strengths and limitations

The study's strengths include: a rigorous study design with a large, randomly selected sample from a cross-sectional, population-based study of fatiguing illness; and the careful clinical determination of groups, selection of comparison measures, report of reading levels of the instrument, and correction of p-values for multiple testing.

This study has several limitations. Our existing data did not allow us to conduct test-retest reliability of the MFI-20. Further studies might be needed to explore test-retest reliability of MFI-20 in fatiguing illness. Another limitation is external validity/generalizability. While the study employed random sampling, the population was limited to an adult population in Georgia and could therefore differ from results that might be obtained from implementing the same study design in other regions due to the effect of regional lifestyle. Nonetheless, previous studies on MFI-20 have not identified the effect of regional lifestyle in their study populations. Our cross-sectional data precluded us from examining responsiveness (ability of the MFI-20 to detect clinically important changes over time) and obviates the possibility of eventually examining responsiveness differences due to treatments. Longitudinal studies are needed to determine minimal clinically important differences (MCIDs) of the MFI-20 subscales in fatiguing illness.

In this study, we applied a 0.01 alpha level of statistical significance to adjust for multiple testing instead of the popular standard level of 0.05. This increases our confidence in the associations that were determined to be of statistical significance but also increases the risk of failing to reject a false null hypothesis (a Type II error), and so results in less statistical power. However, the statistically significant results observed in this study are of practical significance. For example, the group mean differences in our study are greater than the generic MCID of two points in the MFI-20 subscales in Purcell's study [37]. The possibility of a Type II error should, however, be considered.

## Conclusions

This study further demonstrates that the MFI-20 appears to be a valid and reliable measure of chronically unwell and well populations with a stable multidimensional factorial structure. It also suggests that the MFI-20 could indeed be a useful tool for further investigation of generic functional impairment and a complementary diagnostic tool to depression-specific and anxiety-specific instruments in fatiguing illnesses such as chronic fatigue syndrome.

## Competing interests

The authors declare that they have no competing interests.

## Authors' contributions

JML contributed to the conception of the manuscript, had primary responsibility for data processing, statistical analyses, and interpretation of the data, and wrote the manuscript. DJB contributed to intellectual input to data interpretation, streamlining the introduction, and revising the manuscript. EM contributed to data interpretation and critically revised the manuscript. EN contributed to tabulating the results and literature search, and revised the manuscript. RB contributed to intellectual input in the discussion section and revised the manuscript. WCR was Principal Investigator of the source study, collaborating with others in designing the study, writing the protocol, supervising field work, interpretation of the data, and critically revising the manuscript. All authors have read and approved the final manuscript.

## Supplementary Material

Additional file 1 The supplementary materials include the sex and age-specific norms of five MFI-20 subscales and the convergent validity by three study groups (CFS-like, chronically unwell, and well). Click here for file
