# Supplementary material for: Further validation of the Multidimensional Fatigue Inventory in a US adult population sample
Source: Popul Health Metr. 2009 Dec 15;7:18. doi: 10.1186/1478-7954-7-18 (PMC2801470; doi:10.1186/1478-7954-7-18)
Supplement: Additional file 1 — The supplementary materials include the sex and age-specific norms of five MFI-20 subscales and the convergent validity by three study groups (CFS-like, chronically unwell, and well). [file 1478-7954-7-18-S1.DOC]

**Table S1.** Descriptive statistics for the five MFI-20 scales by sex.

|  | All  (n=783) | Female  (n=597) | Male  (n=186) |
| --- | --- | --- | --- |
| **General Fatigue** |  |  |  |
| **Mean** | **12.90** | **13.24** | **11.79** |
| **SD** | **4.68** | **4.71** | **4.44** |
| 25% | 9.00 | 9.00 | 8.00 |
| 50% | 14.00 | 14.00 | 12.00 |
| 75% | 17.00 | 17.00 | 16.00 |
| Range | 4-20 | 4-20 | 4-20 |
| **Physical Fatigue** |  |  |  |
| **Mean** | **10.85** | **11.12** | **10.00** |
| **SD** | **4.36** | **4.37** | **4.25** |
| 25% | 7.00 | 7.00 | 6.00 |
| 50% | 11.00 | 11.00 | 10.00 |
| 75% | 14.00 | 14.00 | 13.00 |
| Range | 4-20 | 4-20 | 4-20 |
| Reduced Activity |  |  |  |
| Mean | 9.25 | 9.30 | 9.07 |
| SD | 4.16 | 4.26 | 3.84 |
| 25% | 6.00 | 6.00 | 6.00 |
| 50% | 8.00 | 8.00 | 8.00 |
| 75% | 12.00 | 12.00 | 12.00 |
| Range | 4-20 | 4-20 | 4-20 |
| Reduced Motivation |  |  |  |
| Mean | 9.58 | 9.67 | 9.30 |
| SD | 3.90 | 4.04 | 3.38 |
| 25% | 6.00 | 6.00 | 7.00 |
| 50% | 9.00 | 9.00 | 9.00 |
| 75% | 12.00 | 13.00 | 11.00 |
| Range | 4-20 | 4-20 | 4-20 |
| **Mental Fatigue** |  |  |  |
| **Mean** | **10.95** | **11.15** | **10.30** |
| **SD** | **4.54** | **4.66** | **4.10** |
| 25% | 7.00 | 7.00 | 7.00 |
| 50% | 11.00 | 11.00 | 10.00 |
| 75% | 14.00 | 15.00 | 13.00 |
| Range | 4-20 | 4-20 | 4-20 |

**Table S2.** Descriptive statistics for the 5 MFI-20 scales by sex and four age groups.

|  | Female |  |  |  | Male |  |  |  |
| --- | --- | --- | --- | --- | --- | --- | --- | --- |
|  | 18-29 yrs  (n=73) | 30-39 yrs  (n=117) | 40-49 yrs  (n=214) | 50-59 yrs  (n=193) | 18-29 yrs  (n=21) | 30-39 yrs  (n=30) | 40-49 yrs  (n=69) | 50-59 yrs  (n=66) |
| General Fatigue |  |  |  |  |  |  |  |  |
| Mean | 13.79 | 13.41 | 13.45 | 12.70 | 12.43 | 11.23 | 12.14 | 11.47 |
| SD | 4.38 | 4.29 | 4.78 | 4.96 | 3.99 | 4.17 | 4.39 | 4.77 |
| 25% | 12.00 | 10.00 | 9.00 | 8.00 | 9.00 | 8.00 | 9.00 | 7.00 |
| Median | 15.00 | 14.00 | 14.50 | 13.00 | 13.00 | 12.00 | 13.00 | 12.00 |
| 75% | 17.00 | 17.00 | 17.00 | 17.00 | 16.00 | 15.00 | 16.00 | 16.00 |
| Range | 4-20 | 4-20 | 4-20 | 4-20 | 6-18 | 4-17 | 4-20 | 4-20 |
| Physical Fatigue |  |  |  |  |  |  |  |  |
| Mean | 9.64 | 10.92 | 11.59 | 11.27 | 9.05 | 8.70 | 10.67 | 10.20 |
| SD | 3.59 | 4.10 | 4.52 | 4.53 | 3.15 | 2.68 | 4.52 | 4.71 |
| 25% | 6.00 | 8.00 | 8.00 | 7.00 | 6.00 | 6.00 | 7.00 | 6.00 |
| Median | 10.00 | 11.00 | 12.00 | 11.00 | 10.00 | 10.00 | 10.00 | 9.50 |
| 75% | 12.00 | 14.00 | 15.00 | 15.00 | 11.00 | 11.00 | 14.00 | 14.00 |
| Range | 4-17 | 4-20 | 4-20 | 4-20 | 4-14 | 4-14 | 4-20 | 4-20 |
| Reduced Activity |  |  |  |  |  |  |  |  |
| Mean | 8.51 | 8.64 | 9.70 | 9.57 | 9.10 | 7.47 | 9.57 | 9.27 |
| SD | 3.48 | 3.94 | 4.53 | 4.34 | 2.95 | 2.34 | 4.19 | 4.12 |
| 25% | 6.00 | 6.00 | 6.00 | 6.00 | 7.00 | 6.00 | 6.00 | 6.00 |
| Median | 8.00 | 8.00 | 9.00 | 9.00 | 9.00 | 7.50 | 9.00 | 8.00 |
| 75% | 11.00 | 11.00 | 13.00 | 12.00 | 10.00 | 9.00 | 12.00 | 12.00 |
| Range | 4-19 | 4-20 | 4-20 | 4-20 | 4-15 | 4-13 | 4-20 | 4-20 |
| Reduced Motivation |  |  |  |  |  |  |  |  |
| Mean | 8.59 | 9.36 | 9.97 | 9.94 | 8.43 | 8.67 | 9.99 | 9.14 |
| SD | 3.35 | 3.42 | 4.24 | 4.34 | 2.99 | 3.17 | 3.57 | 3.33 |
| 25% | 6.00 | 7.00 | 6.00 | 6.00 | 6.00 | 6.00 | 8.00 | 6.00 |
| Median | 8.00 | 9.00 | 10.00 | 10.00 | 8.00 | 8.50 | 10.00 | 9.00 |
| 75% | 11.00 | 12.00 | 13.00 | 13.00 | 9.00 | 11.00 | 12.00 | 12.00 |
| Range | 4-17 | 4-17 | 4-20 | 4-20 | 4-15 | 4-15 | 4-20 | 4-20 |
| Mental Fatigue |  |  |  |  |  |  |  |  |
| Mean | 11.38 | 11.27 | 11.53 | 10.58 | 11.33 | 9.97 | 10.45 | 9.95 |
| SD | 4.75 | 4.63 | 4.70 | 4.57 | 3.81 | 3.86 | 4.18 | 4.23 |
| 25% | 8.00 | 7.00 | 8.00 | 6.00 | 8.00 | 7.00 | 8.00 | 7.00 |
| Median | 11.00 | 11.00 | 11.50 | 11.00 | 11.00 | 10.50 | 10.00 | 9.00 |
| 75% | 15.00 | 15.00 | 16.00 | 13.00 | 14.00 | 12.00 | 13.00 | 12.00 |
| Range | 4-20 | 4-20 | 4-20 | 4-20 | 6-19 | 4-18 | 4-20 | 4-20 |

**Table S3.** Relationships of the MFI-20^†^ subscales to depression, anxiety, and functional impairment in CFS-like group.

|  | MFI-20 | | | | | |
| --- | --- | --- | --- | --- | --- | --- |
|  | General  Fatigue | Physical  Fatigue | Reduced  Activity | Reduced  Motivation | Mental  Fatigue | Total  Score |
|  |  |  |  |  |  |  |
| SF-36 |  |  |  |  |  |  |
| Physical Functioning | -0.27 | **-0.57** | -0.43 | -0.33 | § | -0.39 |
| Role Physical | -0.28 | -0.41 | -0.31 | -0.24 |  | -0.28 |
| Bodily Pain | -0.16 | -0.39 | -0.22 | -0.16 |  | -0.16 |
| Social Functioning | -0.30 | -0.37 | -0.38 | -0.46 | -0.28 | -0.47 |
| Mental Health | -0.24 | -0.20 | -0.24 | -0.38 | -0.39 | -0.42 |
| Role Emotional | -0.21 | -0.15 | -0.18 | -0.23 | -0.33 | -0.32 |
| Vitality | **-0.55** | -0.43 | -0.37 | **-0.54** | -0.23 | **-0.55** |
| General Health | -0.43 | **-0.56** | -0.36 | -0.37 | -0.26 | -0.47 |
| PCS^‡^ | -0.28 | **-0.57** | -0.37 | -0.24 |  | -0.29 |
| MCS | -0.26 | -0.16 | -0.25 | -0.40 | -0.41 | -0.44 |
|  |  |  |  |  |  |  |
| SDS |  |  |  |  |  |  |
| SDS Index | 0.31 | 0.26 | 0.34 | **0.50** | 0.38 | **0.51** |
|  |  |  |  |  |  |  |
| STAI |  |  |  |  |  |  |
| State-Anxiety Score | 0.17 | 0.14 | 0.19 | 0.29 | 0.31 | 0.32 |
| Trait-Anxiety Score | 0.24 | 0.16 | 0.25 | 0.39 | 0.42 | 0.43 |
|  |  |  |  |  |  |  |

^§^ Nonsignificant values (p-value > 0.01 ) were not reported in the table.

Absolute correlation coefficients of 0.5 to 1.0 [35] are considered high correlations, in bold.

^†^ Multidimensional Fatigue Inventory (MFI-20), the Medical Outcomes Survey Short Form-36 (SF-36), the Zung Self-Rating Depression Scale (SDS), and the Spielberger State-Trait Anxiety Inventory (STAI)

^‡^ PCS: Physical Component Summary; MCS: Mental Component Summary

**Table S4.** Relationship of MFI-20^†^ subscales to depression, anxiety, and functional impairment in chronically unwell group

|  | MFI-20 | | | | | |
| --- | --- | --- | --- | --- | --- | --- |
|  | General  Fatigue | Physical  Fatigue | Reduced  Activity | Reduced  Motivation | Mental  Fatigue | Total  Score |
|  |  |  |  |  |  |  |
| SF-36 |  |  |  |  |  |  |
| Physical Functioning | -0.26 | -0.47 | -0.33 | -0.26 | -0.15 | -0.33 |
| Role Physical | -0.34 | -0.47 | -0.35 | -0.29 | -0.20 | -0.39 |
| Bodily Pain | -0.22 | -0.36 | -0.20 |  |  | -0.21 |
| Social Functioning | -0.33 | -0.36 | -0.36 | -0.44 | -0.29 | -0.47 |
| Mental Health | -0.41 | -0.33 | -0.35 | -0.48 | -0.38 | -0.54 |
| Role Emotional | -0.26 | -0.32 | -0.31 | -0.30 | -0.33 | -0.40 |
| Vitality | **-0.74** | **-0.57** | -0.48 | **-0.57** | -0.30 | **-0.70** |
| General Health | -0.40 | **-0.54** | -0.34 | -0.29 | -0.11 | -0.38 |
| PCS^‡^ | -0.28 | **-0.52** | -0.29 | -0.16 |  | -0.25 |
| MCS^‡^ | -0.43 | -0.33 | -0.39 | **-0.52** | -0.40 | **-0.58** |
|  |  |  |  |  |  |  |
| SDS |  |  |  |  |  |  |
| SDS Index | 0.44 | 0.40 | 0.36 | **0.51** | 0.41 | **0.58** |
|  |  |  |  |  |  |  |
| STAI |  |  |  |  |  |  |
| State-Anxiety Score | 0.28 | 0.24 | 0.20 | 0.37 | 0.39 | 0.42 |
| Trait-Anxiety Score | 0.37 | 0.30 | 0.32 | 0.47 | 0.42 | **0.53** |
|  |  |  |  |  |  |  |

^§^ Nonsignificant values (p-value > 0.01) were not reported in the table.

Absolute correlation coefficients of 0.5 to 1.0 [35] are considered high correlations, in bold.

^†^ Multidimensional Fatigue Inventory (MFI-20), the Medical Outcomes Survey Short Form-36 (SF-36), the Zung Self-Rating Depression Scale (SDS), and the Spielberger State-Trait Anxiety Inventory (STAI)

^‡^ PCS: Physical Component Summary; MCS: Mental Component Summary

**Table S5.** Relationship of MFI-20^†^ subscales to depression, anxiety, and functional impairment in well control group.

|  | MFI-20 | | | | | |
| --- | --- | --- | --- | --- | --- | --- |
|  | General  Fatigue | Physical  Fatigue | Reduced  Activity | Reduced  Motivation | Mental  Fatigue | Total  Score |
|  |  |  |  |  |  |  |
| SF-36 |  |  |  |  |  |  |
| Physical Functioning | -0.38 | **-0.53** | -0.45 | -0.44 | -0.31 | **-0.50** |
| Role Physical | -0.38 | -0.34 | -0.19 | -0.36 | -0.23 | -0.38 |
| Bodily Pain | -0.27 | -0.29 |  | -0.19 |  | -0.23 |
| Social Functioning | -0.33 | -0.30 | -0.27 | -0.38 | -0.27 | -0.40 |
| Mental Health | -0.40 | -0.34 | -0.26 | -0.40 | -0.35 | -0.46 |
| Role Emotional | -0.35 | -0.28 | -0.26 | -0.34 | -0.29 | -0.40 |
| Vitality | **-0.69** | **-0.59** | **-0.50** | **-0.59** | -0.42 | **-0.71** |
| General Health | **-0.58** | **-0.69** | **-0.53** | **-0.50** | -0.35 | **-0.63** |
| PCS^‡^ | -0.42 | **-0.54** | -0.35 | -0.38 | -0.23 | -0.45 |
| MCS^‡^ | -0.44 | -0.34 | -0.31 | -0.44 | -0.36 | **-0.51** |
|  |  |  |  |  |  |  |
| SDS |  |  |  |  |  |  |
| SDS Index | 0.34 | 0.31 | 0.24 | 0.42 | 0.40 | 0.45 |
|  |  |  |  |  |  |  |
| STAI |  |  |  |  |  |  |
| State-Anxiety Score | 0.30 | 0.29 | 0.31 | 0.40 | 0.30 | 0.42 |
| Trait-Anxiety Score | 0.39 | 0.30 | 0.30 | 0.43 | 0.39 | 0.49 |
|  |  |  |  |  |  |  |

^§^ Nonsignificant values (p-value > 0.01) were not reported in the table.

Absolute correlation coefficients of 0.5 to 1.0 [35] are considered high correlations, in bold.

^†^ Multidimensional Fatigue Inventory (MFI-20), the Medical Outcomes Survey Short Form-36 (SF-36), the Zung Self-Rating Depression Scale (SDS), and the Spielberger State-Trait Anxiety Inventory (STAI)

^‡^ PCS: Physical Component Summary; MCS: Mental Component Summary
